# External causes of death in younger than 18 years old in Portugal in the last 10 years - a retrospective analysis

**DOI:** 10.1007/s12024-025-00997-7

**Published:** 2025-03-27

**Authors:** Carlota Jardim Gomes, Marta Heitor, Joana Albuquerque, Ana Rita Inácio

**Affiliations:** 1 Delegação do Sul do Instituto Nacional de Medicina Legal e Ciências Forenses, Lisboa, Portugal; 2 Faculdade de Medicina, Universidade de Lisboa, Lisboa, Portugal

**Keywords:** Pediatric mortality, External causes of death, Violent deaths, Child injury prevention

## Abstract

Pediatric mortality from external causes has been a worldwide concern in the last decades. In particular, the prevalence of accidental deaths is a key concern, especially traffic accidents. This is retrospective study based on autopsy reports of violent deaths in individuals younger than 18 years from 2014 to 2023 in Portugal, aimed at providing valuable insight in order to help formulate preventive strategies. There were 554 pediatric deaths due to exogenous causes, with a predominance of males (68,95%). Adolescents were the most prevalent age group. The leading cause of death was land transport injury (38,27%). Asphyxia-related deaths were predominant in younger age groups. Accidental deaths accounted for 76,71% of all cases. Preventable injury-related causes continue to be a major contributor to child mortality. The inconsistent mortality rates from various mechanisms emphasize the necessity for targeted and effective preventive measures. Above all, land transport accidents seem to be an issue in need of prompt intervention.

## Introduction

As stated by The United Nations Convention on the Rights of the Child (1989), “a child means every human being below the age of eighteen years unless under the law applicable to the child, majority is attained earlier” [1]. The pediatric population is especially vulnerable and require a greater number of measures to ensure a healthy development in a safe environment.

Pediatric mortality has been regarded as a comprehensive and accessible indicator to assess the health, environmental, and social circumstances of children. In the last decades, progress has been made to decrease child mortality rates, particularly due to infectious and oncological diseases [2–4], owing to the ongoing medical advancements. As a result, there has been a shift towards increased mortality from exogenous causes.

Traumatic injuries are the leading cause of death among individuals aged 5–17 years in the European region [5], with a higher incidence observed in females [4, 6–11]. The causes of these injuries may vary according to the population’s socioeconomic status [5, 10].

Recent national data indicate that external causes account for 28% of deaths between 1 and 4 years old and 55% of deaths between 15 and 19 years old [12]. In a population-based study, from 1987 to 2011, trauma accounted for 23.7% of all deaths in children aged 0–17 years, which highlights the significant impact of external causes on child mortality in Portugal [13].

The small stature, fragility, and inability to recognize danger make children more susceptible to accidents [14]. Road traffic injuries are a notable area of concern, accounting for an estimated 262 000 deaths in people aged 0 to 19 annually [15].

Furthermore, several studies have reported an increase in pediatric injuries during the COVID-19 pandemic. Intentional injuries, in particular, have been linked to greater vulnerability during times of external stress [16–18].

Suicide is rare in young children, since, in most cases, a well-defined notion of death is not fully established until preadolescence [19, 20]. Thus, most studies show an increase in suicides with age [4, 6–8]. Significant risk factors for suicide in later childhood include mental health disorders like depression and anxiety, especially when combined with substance use [21, 22].

The United Nations Office on Drugs and Crime (UNODC) estimated that 205,153 children (ages 0 to 14) were murdered worldwide between 2008 and 2017 [23]. As reported by Fujiwara T. et al. [24] and by Stöckl, H. et al. [25], these crimes are mainly perpetrated by a family member or a caretaker.

Further data from UNODC revealed that boys aged 15 to 17 are the pediatric group most affected by homicide globally, with their homicide rate in 2016 being about five times higher than that of girls in the same age group [23]. However, infanticide remains a significant concern, with rates in the U.S. reaching as high as 9.2 per 100,000 population in 2000 [23].

To date, the number of published reports of children and adolescents’ deaths due to external causes is scarce. The aim of this study is to characterize and assess the evolution of external causes of death in individuals 17 years old or younger, in the last 10 years (2014 to 2023), in Portugal, using data from autopsy reports carried out at the National Institute of Legal Medicine and Forensic Sciences.

By providing a detailed analysis, this study seeks to help guide the development of effective preventive measures. Despite the preventable nature of this matter, public awareness and political commitment to reducing child deaths by exogenous causes remain insufficient.

## Methods

A retrospective study carried out by consulting autopsy reports of the National Institute of Legal Medicine and Forensic Sciences in Portugal. The data inclusion criteria were: (a) individuals 17 years old or younger; (b) manner of death was classified as violent (accidental, suicidal, homicidal or with undetermined intent); (c) in the last 10 years (2014–2023).

Data was gathered from 1,015 autopsy reports using Microsoft Excel™, of which 442 cases were excluded due to a data registration error of individuals with or above 18 years of age. Additionally, 19 fetal deaths were excluded.

The subjects were categorized into five groups, according to age: newborns (up to 1 month old), infants (1–11 months old), toddlers (1–3 years old), children (4–12 years old), and adolescents (13–17 years old).

## Results

**Fig. 1:**
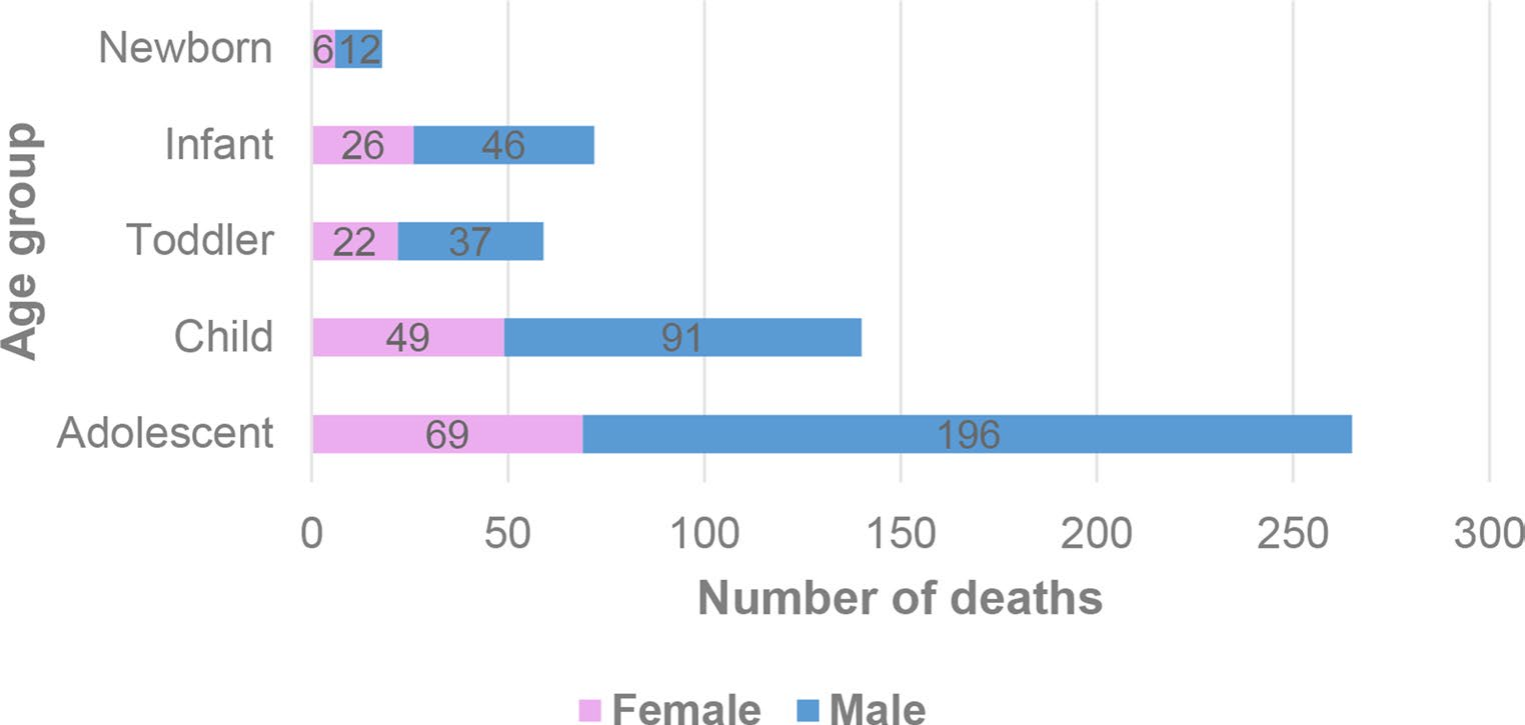
Number of deaths by age group and gender (*n* = 554)

Of the 554 analyzed cases, 382 (68.95%) were male, while 172 (31.05%) were female, with males being more prevalent in every age group. Newborn deaths corresponded to 18 cases (3,25%), infants to 72 cases (13,00%), toddlers to 59 cases (10,65%), children to 140 cases (25,27%) and adolescents to 265 cases (47,83%) (Fig. 1).

**Fig. 2:**
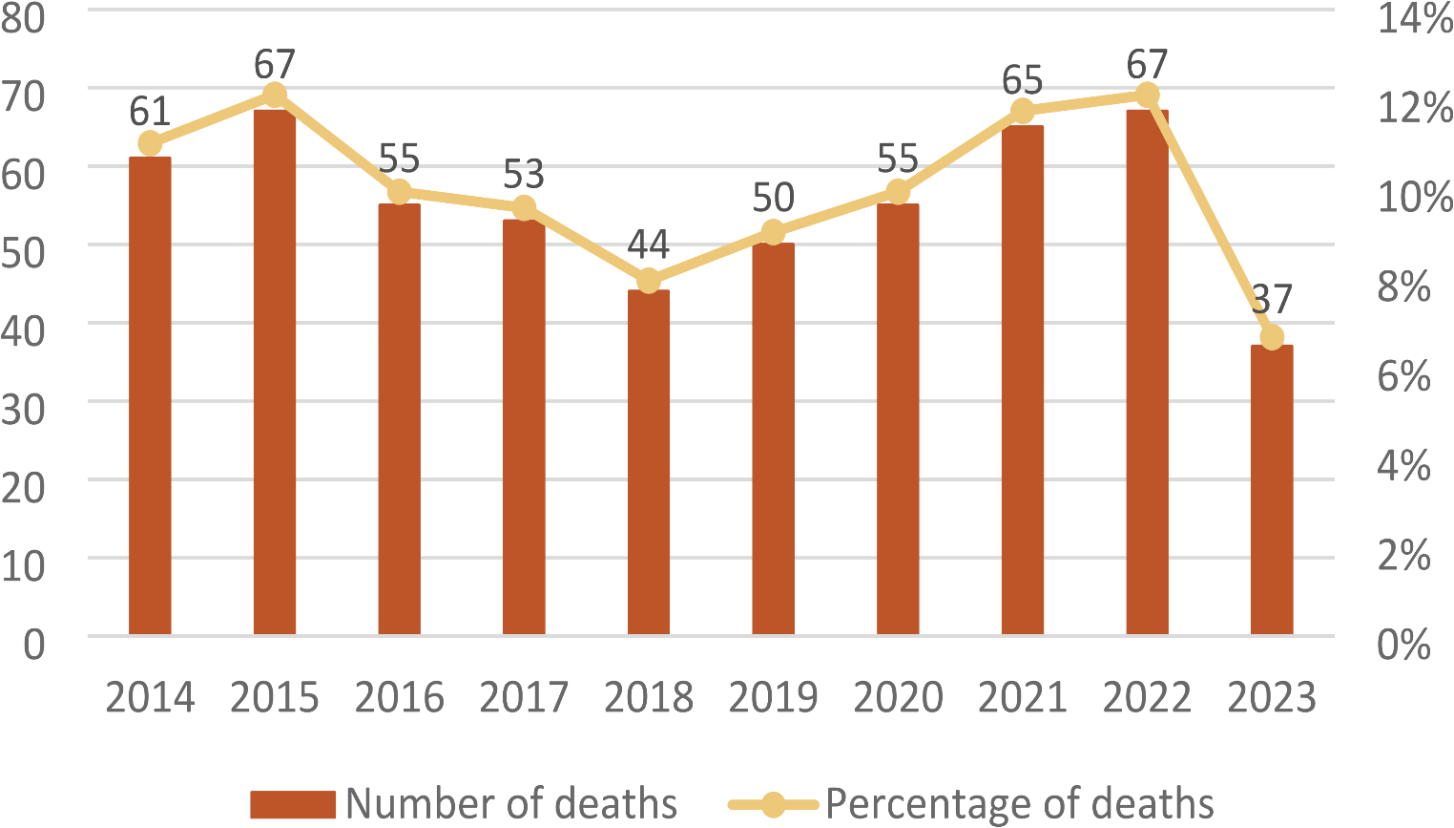
Number and percentage of deaths by year (*n* = 554)

Throughout the examined period, there was a median of 55 deaths annually. As illustrated in Fig. 2, there was an increase in the number of violent deaths in 2014–2015 and in 2021–2022, while in 2023 this number reached its lowest point. During the COVID-19 pandemic (2020–2022), the number of pediatric deaths increased from 147 to 187 compared to the three preceding years, with a slight rise in suicides, from 17 to 21 cases.

**Fig. 3:**
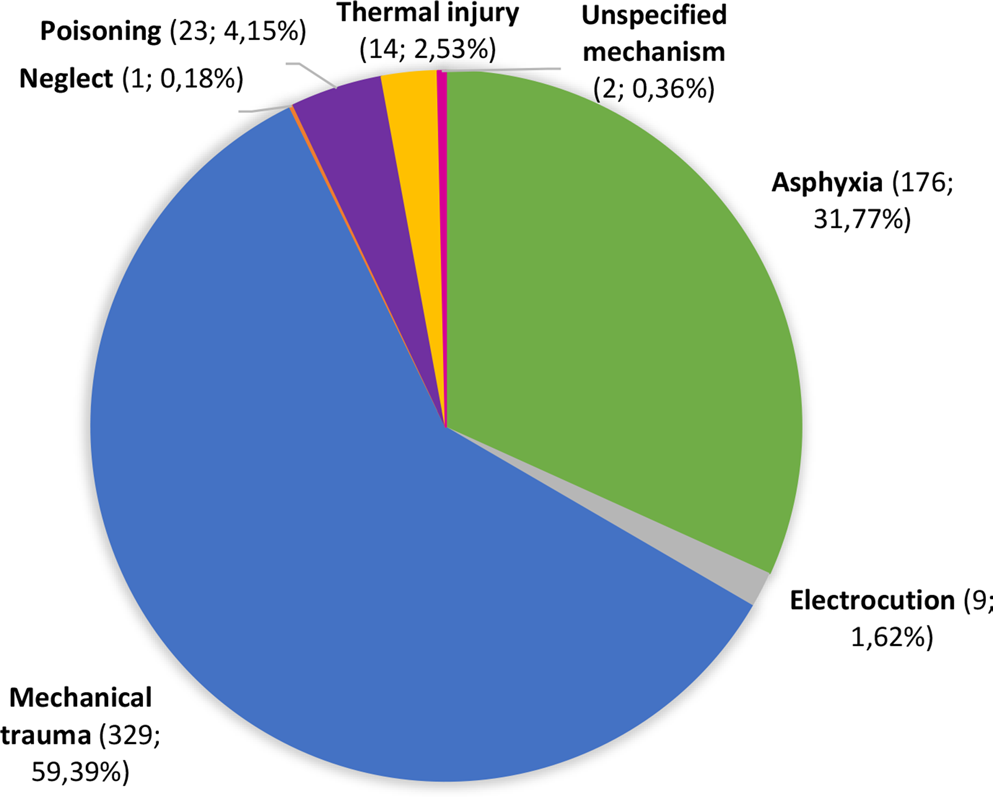
Main causes of deaths (*n* = 554)

Most deaths were caused by mechanical trauma (59.39%) or asphyxia (31.77%) (Fig. 3).

The mechanical trauma category includes various types of injury mechanisms, such as blunt force and penetrating trauma (Table 1). Deaths labeled as resulting from an ‘Unspecified mechanism or event’, in Table 1, refer to cases where the mechanism or cause of traumatic lesions was not detailed in the autopsy report, usually due to missing circumstantial information.

**Table 1:** Distribution of deaths by mechanical trauma (*n* = 329)

Deaths by mechanical trauma	Number of cases
**Unspecified mechanism**	**8 (2,43%)**
**Penetrating trauma**	**18 (5,47%)**
Unspecified event	2 (0,61%)
Animal bite	2 (0,61%)
Gunshot wound	5 (1,52%)
Sharp object	9 (2,73%)
**Blunt force trauma**	**303 (92**,**10%)**
Aquatic transport	2 (0,61%)
Sequelae of labor dystocia	3 (0,91%)
Shaken Baby Syndrome	4 (1,22%)
Unspecified event	4 (1,22%)
Assault	5 (1,52%)
Other specified event	5 (1,52%)
Railway transport	22 (6,68%)
Fall	46 (13,98%)
Land transport	212 (64,44%)
**Total**	**329 (100**,**00%)**

Asphyxia-related deaths were primarily caused by accidental drowning (44,32%). Airway obstruction (e.g. food or gastric content, blood, foreign body) and hanging were also common causes (Table 2).

**Table 2:** Distribution of deaths by asphyxia (*n* = 176)

Deaths by asphyxia	Number of cases
Manual Strangulation	3 (1,70%)
Strangulation	3 (1,70%)
Traumatic asphyxia	4 (2,27%)
Smothering	4 (2,27%)
Hanging	33 (18,75%)
Airway obstruction	51 (28,98%)
Drowning	78 (44,32%)
**Total Geral**	**176 (100%)**

**Fig. 4:**
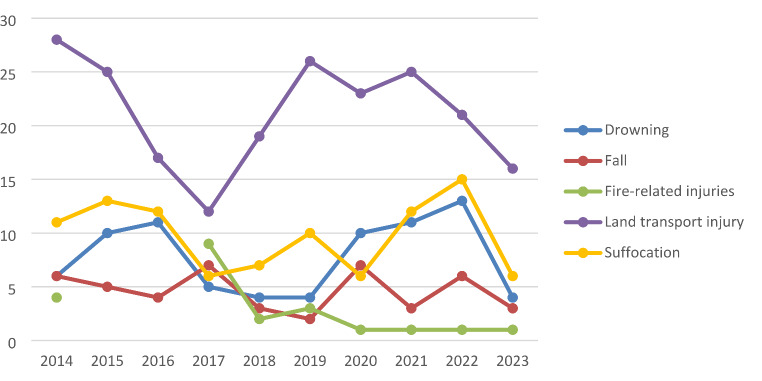
Number of deaths per year for the five leading causes of death (*n* = 456)

In the last decade, the five leading causes of death were land transport injury (38,27%), asphyxia by suffocation (17,69%; e.g., smothering, obstruction of the airway, hanging, strangulation), asphyxia by drowning (14,08%), fall (8,30%) and fire-related injuries (3,97%). Of these mechanisms, only fire-related injuries showed a downwards tendency over the examined period (Fig. 4).

**Fig. 5:**
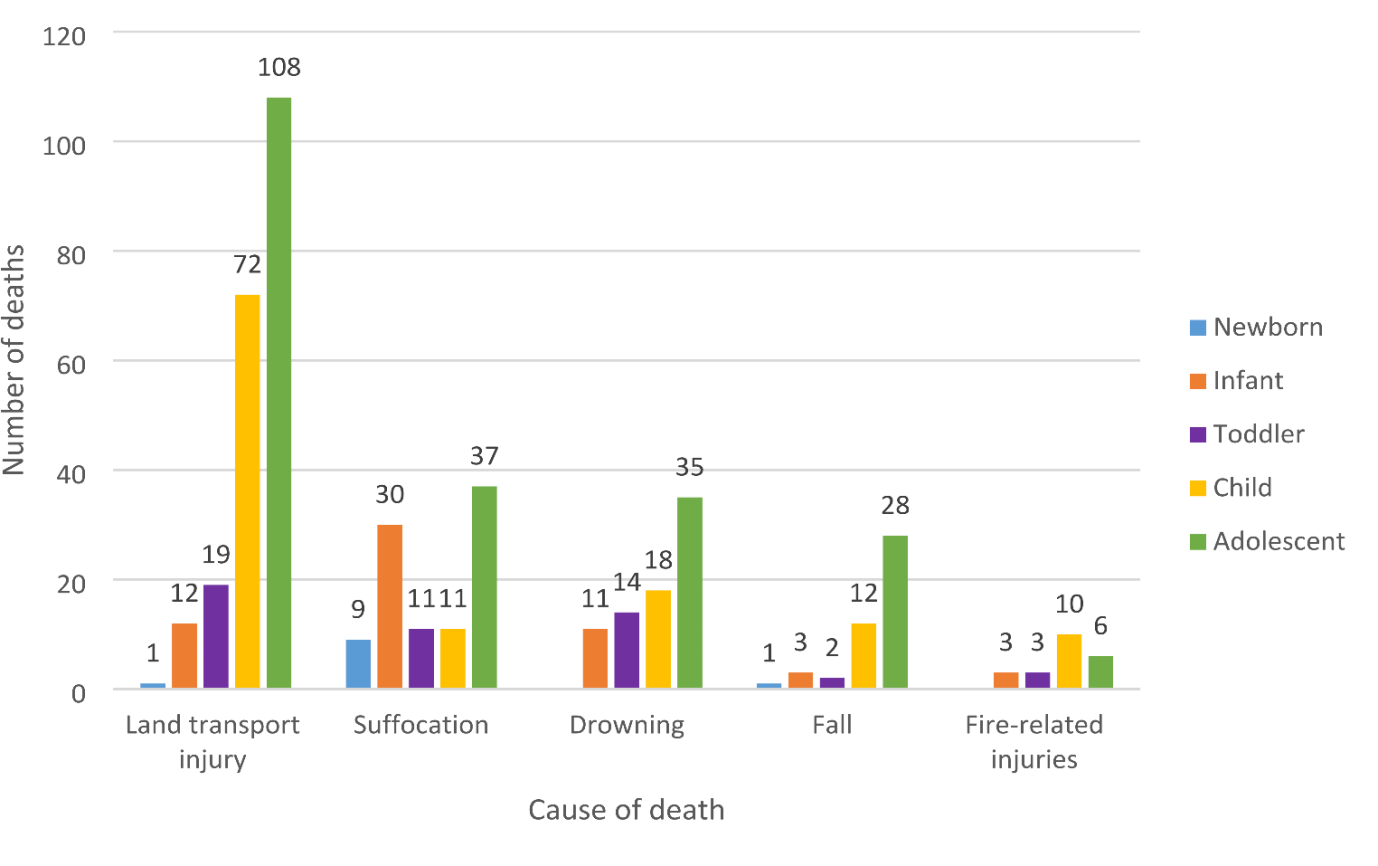
Number of deaths per age group for the five leading causes of death (*n* = 456)

For older age groups (adolescents and children), land transport injuries are the most prevalent cause of death, suggesting that traffic safety for these age groups is a major concern. Moreover, age seems to correlate with an increased risk of these type of injuries (Fig. 5).

Suffocation was the leading cause of death in younger age groups (newborns and infants) (Fig. 5), primarily due to accidents, whereas in adolescents, it was frequently linked to suicidal intent.

The risk of fatal drowning increased with age (Fig. 5), with most of these deaths resulting from accidents. Fall-related deaths were most common in adolescents (Fig. 5), 46.43% of which were classified as suicides. Fire-related injuries were most frequently observed in children, accounting for 45.45% of cases (Fig. 5).

**Fig. 6:**
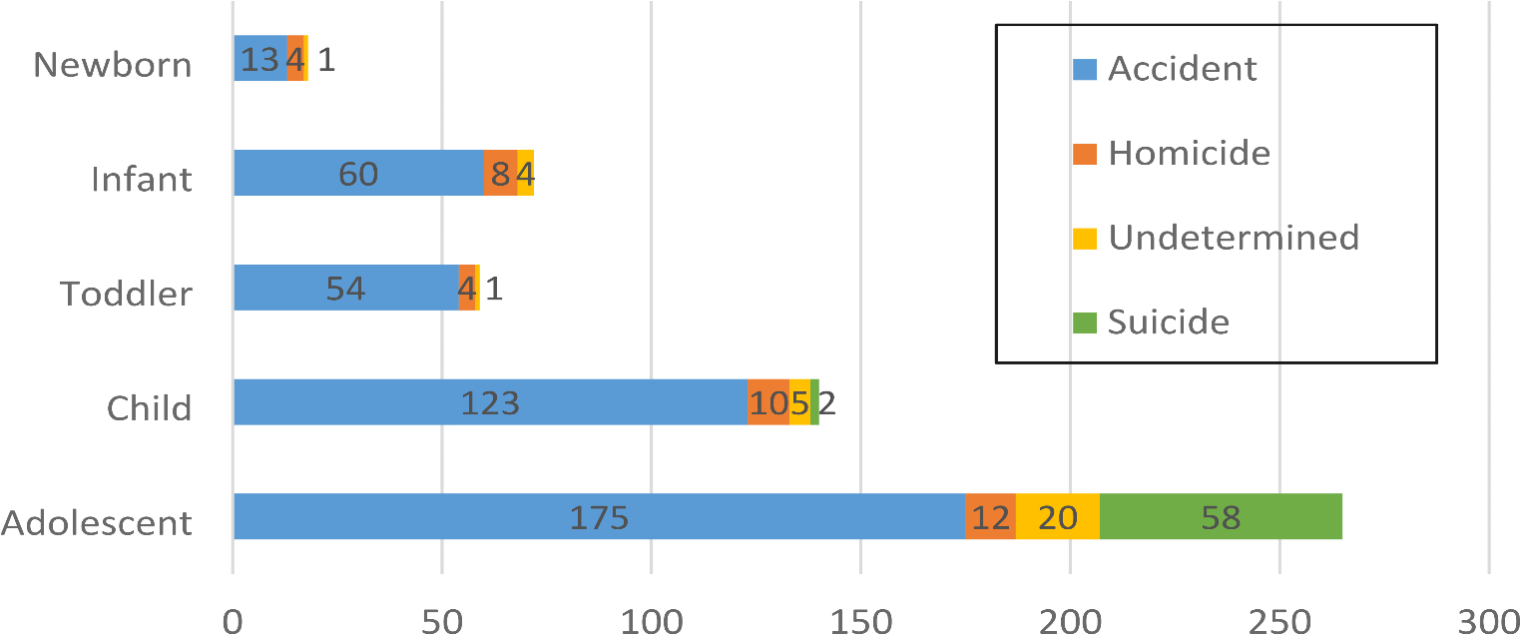
Distribution of manner of death by age group (*n* = 554)

When we examined all 554 deaths according to intent, accidents were the most common manner of injury-related death (76,71%) (Fig. 6), with a median of 42,5 deaths per year.

Among intentional injuries, suicide was slightly more common (10,83%) than homicide (6,86%) (Fig. 6). The most common method of suicide was hanging (46,67%), whereas homicides were predominantly carried out through penetrating (31,58%) or blunt force (28,95%) trauma.

## Discussion

This study provides an analysis and description of external causes of death in the pediatric population, in Portugal, between 2014 and 2023.

The leading causes of pediatric deaths in this period were **land transport injuries**,** suffocation**,** fire-related injuries**,** and falls**.

The study showed a **predominance of male adolescents** among those who died from external causes, aligning with findings from multiple studies [4, 7, 10, 11, 26]. This higher vulnerability in male adolescents may be related to factors such as impulsivity and inquisitive nature, as well as a greater exposure to risk behaviors [26–28].

Over this period, there was **no consistent downward trend** in pediatric deaths from exogenous causes. A notable decline in deaths was observed in 2023, which may be partially attributed to the possibility that some autopsy reports were not included in the database at the time of data collection, rather than a true decline in deaths. However, this decline could also be associated with factors such as: improved and efficient safety measures and advancements in medical response to pediatric trauma. Further research would be required to determine the exact reasons for this fluctuation.

The observed increase in pediatric deaths during the COVID-19 pandemic aligns with existing research highlighting the heightened vulnerability of the juvenile population during periods of external stress [16–18]. While the surge in suicides was modest, it reinforces concerns about the long-term psychological impact of the pandemic on younger populations.

These findings may be attributed to several pandemic-related factors, including social isolation, limited access to mental health support, and increased family stressors [16–18]. Disruptions in healthcare services and emergency response systems could have also played a role in delaying critical interventions. Ensuring adherence to treatment for mental health disorders, along with improved mental health support and crisis intervention strategies, is essential for mitigating the impact of such crises on pediatric populations in the future.

Although the overall number and specific causes of death fluctuated over time, there was little to no decline in any cause of traumatic death over the past decade.

**Fire-related injuries** - the only exception to this tendency– peaked in 2017, which correlates with the devastating wildfires in the central region of Portugal (Pedrógão Grande, Leiria), in June of that year, triggered by an intense heatwave, which resulted in the deaths of 6 minors and 60 adults. This underscores the significant impact of environmental factors, such as natural disasters, on pediatric mortality.

Epidemiological data from World Health Organization (WHO) demonstrated a discrepancy in mortality rates by this mechanism between high-income countries, such as Portugal, and low-income countries [5, 29]. The high frequency in young children, as shown in our study, may be explained by a growing curiosity about fire and experiment with matches, lighters or firework, or even the act of copying caregivers’ tasks, like ironing or cooking [5].

Typically, deaths by burn-injuries or carbon monoxide poisoning are strongly connected to unsafe environments and products. Recommended prevention strategies vary from installing smoke alarms and sprinkler systems to the reinforcement of legislation restricting children’s access to fireworks. The development of child-resistant ignition devices could also significantly reduce the number of fires started by children. Additionally, educational programs for children and parents — focused on fire safety and the risks of storing flammable products at home—have proven to be effective [30].

For children over the age of 1, **land transport injury** was the most common cause of traumatic death, a finding supported by WHO data, which ranks road traffic injuries as the leading cause of death among children in the European region [5, 29]. In fact, the high child mortality rate due to land transport injury has been a major concern in Portugal since the 1980s, as the country has one of the highest child mortality rates in Europe [5, 31].

The fluctuating pattern in the number of land traffic injury-related deaths shown in our study (Fig. 4) could be attributed to a combination of multiple factors, such as: the effects of new safety campaigns; the implementation or changes in road safety laws; weather patterns; economic downturns; pandemic restrictions (e.g., travel restrictions during COVID-19); as well as changes in patterns of transport and exposure.

Several risk factors related to deaths from traffic accidents have been identified, such as driving under the influence of alcohol, stress or fatigue, excessive speed, poor road signs or conditions, and factors related to vehicles (inadequate tire, brake and engine maintenance and a lack of efficient airbags) [5, 26]. Over the last decade, measures to improve traffic safety have been implemented, however, these types of deaths have sustained their position as the leading cause of fatal injury, raising concerns about social compliance and the effectiveness of these strategies.

The increase in land transport-related deaths with age observed in our study may be linked to a greater propensity for engaging in risky behaviors. Recent data also shows that distraction by mobile technologies may play a role. A multisite observational study of 34 000 students crossing in school zones found that 1 in 5 high school students were observed to be inattentive using cellular phones or listening to music [32].

For neonates and infants, **suffocation** was the most common mechanism of death, mainly due to airway obstruction by inhalation of food or other objects. This finding is in agreement with study results from the UK [4], US [10], Brazil [26] and Canada [33].

As an initiative to prevent this issue, child health care providers should discuss choking-prevention strategies with parents and school staff. As recommended by the *Canadian Peadiatric Society* [34], it is pertinent to revise national laws related to child products sales, to include known hazards that are not currently regulated, but are associated with fatal incidents.

Portugal has a low **drowning** mortality rate compared to other European countries [5], with accidental drownings (88.31% of cases) being most common. While deaths in younger age groups are usually related to lack of supervision or absence of a pool barrier, in adolescents a common risk factor is alcohol/drug abuse.

A wide selection of effective interventions is recommended in order to reduce the national burden of drowning injuries: placing pool fences or covering pools; supervision of lifeguards and use of appropriate signage in public swimming sites; swimming education in and out of schools; and the use of personal floatation devices.

In contrast, Portugal is considered to have a relatively high mortality rate by **fall-related injuries** [5], which is in accordance with our results. Additionally, a higher prevalence of fall-related deaths among adolescents was observed, primarily of suicidal intent, whereas accidental falls were more common in the other age groups.

The circumstances of injuries produced by this mechanism also differ between ages: infants and toddlers are more likely to fall from stairs or playground structures or by being dropped, whereas children and adolescents mostly fall from balconies, windows or roofs.

In Europe, great strides have been made in terms of preventive measures, particularly regarding environmental and engineering changes. For example, implementing playground standards and installing safety equipment at home (e.g. window guards, stair gates, guardrails on beds) have proven to be useful [5].

According to guidelines from the Portuguese Association for Child Safety, balcony barriers should be at least 110 cm high, with gaps no wider than 9 cm and neither be climbable nor encourage climbing [35].

To further prevent fall-related deaths in children, increased supervision when near high places can help prevent falls. Providing information on childproofing homes and teaching children about the dangers of climbing can be helpful.

## Limitations

The present review should be considered in light of some limitations. One major limitation is the possibility of missing data, either due to cases not being included in the database or misclassification errors.

Additionally, while the study identifies the causes of pediatric deaths, it lacks detailed contextual information, such as family background or mental health history, which could offer a deeper understanding of risk factors.

Another key limitation is that although the study discusses various preventive measures, it does not evaluate their effectiveness over time. It also does not examine obstacles to implementing these strategies, such as socioeconomic disparities, lack of enforcement, or low public awareness.

Moreover, while the study provides valuable insights into pediatric mortality trends in Portugal, it does not explore long-term changes in public health efforts or how advancements in medical care and emergency response have influenced survival rates. Without this analysis, it remains unclear whether the observed fluctuations in mortality are due to improved interventions or other external factors.

## Conclusion

This study provides a better knowledge of the cause and manners of death in the pediatric population, considering both gender and age group differences.

To our knowledge, no nationwide study has assessed recent data on pediatric mortality from external causes, making this the first to examine long-term trends using the most recent data.

Furthermore, these findings can be applied to other high-income countries, particularly in Europe, where similar risk factors and trends in pediatric mortality have been observed.

The study highlights key contributors to pediatric mortality due to exogenous causes, such as land transport injuries, suffocation, fire-related injuries, and falls.

These findings emphasize the urgent need for multisectoral and targeted prevention strategies, such as enhanced traffic safety measures, fire prevention initiatives, choking hazard awareness, and improved home safety regulations. Particular emphasis should be placed on the public health hazard posed by road traffic accidents, reinforcing the requirement for strategies to enhance vehicle, roadway, and driver safety.

Additionally, the observed trends in suicide rates among adolescents during the COVID-19 pandemic highlight the importance of addressing mental health issues in pediatric populations. This reinforces the necessity of strengthened psychological support systems and crisis intervention strategies worldwide.

By providing a comprehensive analysis of pediatric injury-related mortality, this study serves as a foundation for future efforts aimed at reducing preventable childhood deaths. Further research, particularly in other European countries with limited comparative data, would help expand this understanding.

Lastly, while differences in mortality risk across age groups are noted, future studies are required to develop tailored prevention strategies for each stage of childhood and adolescence.

## Key points

1. This study identifies the leading external causes of death among children and adolescents in Portugal: land transport injuries, suffocation, fire-related injuries, drowning and falls.

2. Different age groups face distinct risks, with suffocation being more common in infants, drowning and fire-related injuries in children, and falls in adolescents.

3. There was no consistent downward trend in pediatric deaths over the past decade (2014-2023), indicating that current prevention efforts may not be sufficiently effective.

4. The COVID-19 pandemic influenced pediatric mortality patterns, with an observed rise in adolescent suicides.

5. Despite the preventable nature of many of these deaths, these findings emphasize the importance of targeted prevention strategies.
